# Supplementary material for: Angiopoietin-2 Inhibition of Thrombomodulin-Mediated Anticoagulation—A Novel Mechanism That May Contribute to Hypercoagulation in Critically Ill COVID-19 Patients
Source: Biomedicines. 2022 Jun 6;10(6):1333. doi: 10.3390/biomedicines10061333 (PMC9220312; doi:10.3390/biomedicines10061333)
Supplement: Supplementary file 1 [file biomedicines-10-01333-s001.zip › biomedicines-1696427-supplementary.pdf]

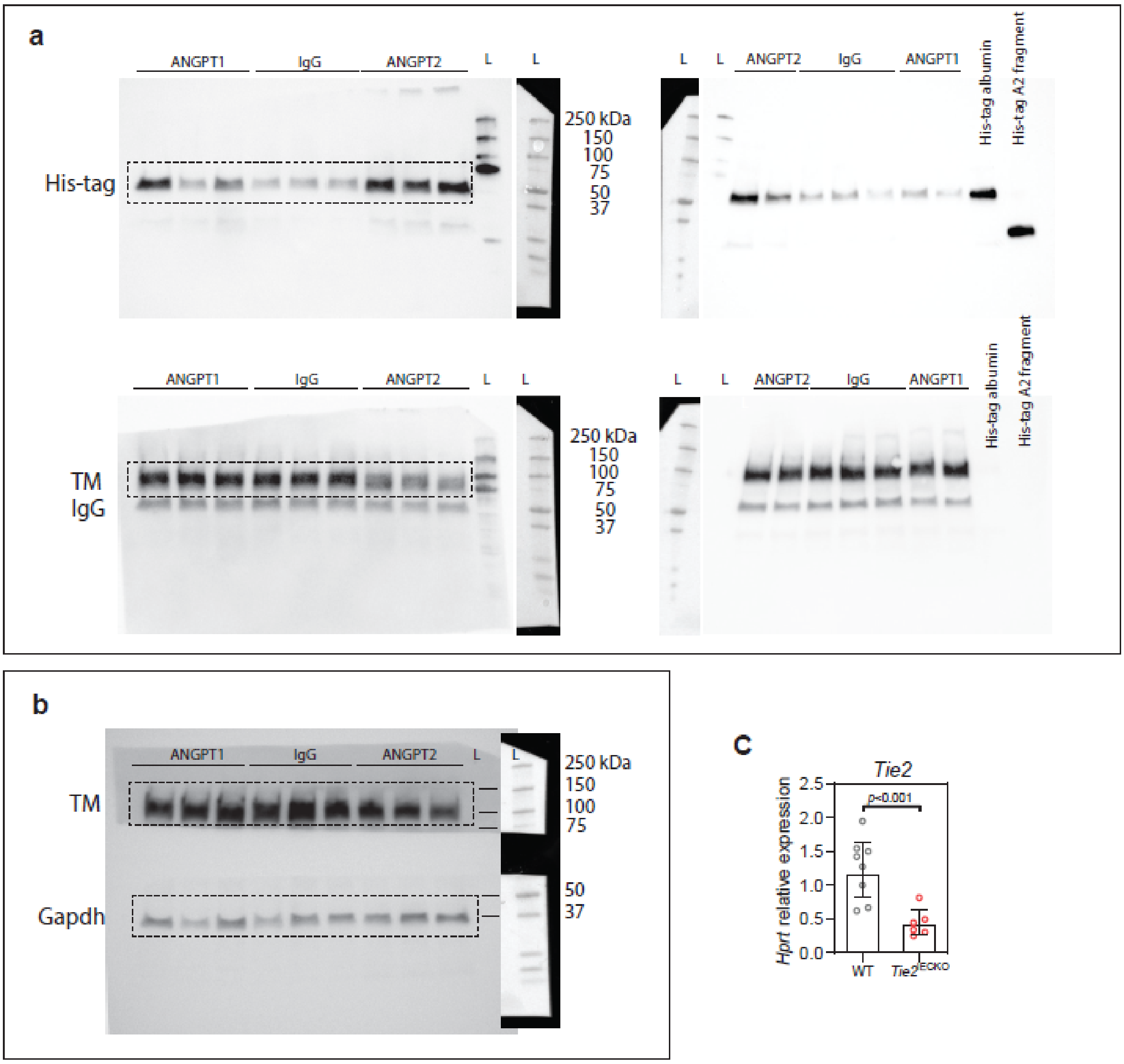

Figure S1. (**a**) Full gel blots of His-tag labelled proteins after immunoprecipitation of TM in lung tissue 15 min after intraperitoneal (i.p.) injection of 80 nmol (high dose) of His-tagged ANGPT2 (A2), ANGPT1 (A1) and IgG. (**b**) Full gel blot of total TM in lung lysates from the same conditions as above. (**c**) Gene expression of Tie2 in kidney lysates in Tie2^{iECKO} mice and littermate controls (WT). Data presented as mean ± 95% CI, statistical comparison with Student's t-test.

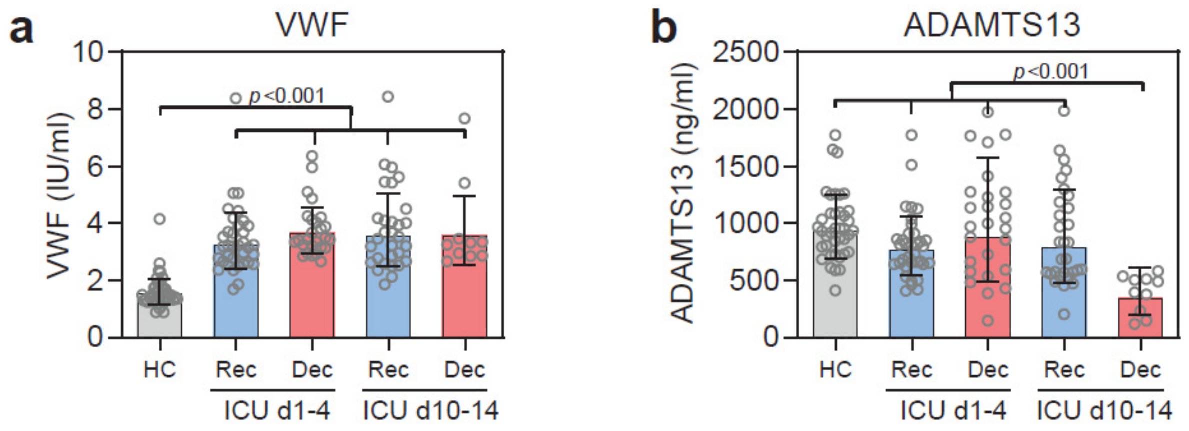

Figure S2. (**a**,**b**) Plasma VWF and ADAMTS13 concentrations for healthy controls (HC), recovered (Rec) and deceased (Dec) patients at day 1-4 and day 10-14 after admission, respectively. Data presented as mean ± 95% CI. Statistical comparison from one-way ANOVA with Bonferroni post hoc.
